# The impact of Sanming’s healthcare reform on medical resource misallocation: based on synthetic control method

**DOI:** 10.3389/fpubh.2025.1671174

**Published:** 2025-10-21

**Authors:** Lin Guo, Chengxin Fan, Chunxiao Yang, Dongping Ma, Kui Sun, Xingang Sang, Zhongming Chen, Hongwei Guo, Wenqiang Yin

**Affiliations:** ^1^School of Management, Shandong Second Medical University, Weifang, Shandong, China; ^2^School of Public Health, Shandong Second Medical University, Weifang, Shandong, China; ^3^Weifang Municipal Health Commission, Weifang, Shandong, China

**Keywords:** healthcare resource misallocation, healthcare reform, synthetic control methods, primary care capacity, health expenditure

## Abstract

**Background:**

The global misallocation of healthcare resources has emerged as a critical impediment to public health. In China, healthcare resources are predominantly concentrated in major cities and high-tier hospitals, while primary care facilities suffer from inadequate capacity, contributing to issues such as “difficult and expensive access to healthcare.” Despite numerous healthcare reforms, significant disparities in resource distribution persist.

**Objective:**

This study seeks to elucidate the causal effects of healthcare system reform on the misallocation of medical resources and to investigate the underlying mechanisms. Focusing on the healthcare reform implemented in Sanming in 2012 as a quasi-natural experiment, the research employs the synthetic control method (SCM) to assess the policy’s impact on resource misallocation.

**Methods:**

The synthetic control method is applied to estimate the causal impact of the Sanming Medical Reform on the misallocation of healthcare resources. By constructing a weighted control group that replicates Sanming’s counterfactual resource allocation trajectory in the absence of the reform, the model controls for covariates such as industrial structure, GDP, and human resources to ensure precise estimation of the policy effect. Data were obtained from the National Bureau of Statistics, local health commissions, and other sources, forming a balanced panel dataset of 203 cities spanning 2007 to 2022.

**Results:**

The analysis reveals that the Sanming Medical Reform markedly reduced the misallocation of healthcare resources. Following the 2012 intervention, Sanming exhibited a substantial decline in misallocation, with the misallocation index decreasing by an average of 0.1412 between 2013 and 2017. Both city-level and time placebo tests confirm that the observed policy effect is statistically significant and not attributable to random variation. Mechanism analysis further indicates that the reform achieved its outcomes by increasing government expenditure on health and refining the structure of health insurance.

**Conclusion:**

The empirical evidence demonstrates that the Sanming Medical Reform effectively alleviated the misallocation of healthcare resources and bolstered primary care capacity through enhanced government spending and optimized health insurance payment structures. These findings offer valuable insights and empirical support for healthcare reforms in China and other countries, paving the way for more equitable and efficient resource allocation.

## 1 Introduction

The global misallocation of healthcare resources has become an ever-more pressing challenge confronting nations worldwide. According to the World Health Organization 2023 report, the Universal Health Coverage service index has improved since 2000. Yet approximately 4.5 billion people still lack access to essential health services and nearly 2 billion face catastrophic out of pocket expenses. This problem also affects middle and high income countries, where stark inequities in healthcare distribution persist (1). China faces similar challenges, with medical resources concentrated primarily in first-tier cities and tertiary hospitals, while the service capabilities of grassroots medical institutions remain relatively weak. Although the government has implemented policies such as tiered diagnosis and treatment to optimize resource allocation, significant imbalances persist, leading to long-standing issues of “difficult and expensive access to healthcare” (2). Therefore, both globally and in China, more effective policy measures are urgently needed to address the issue of healthcare resource misallocation. Medical resource misallocation refers to the spatial and institutional mismatch between healthcare supply and population needs, for example the excessive concentration of beds, physicians and services in tertiary hospitals while primary care facilities remain under-resourced. This form of misallocation undermines both equity and system efficiency and is a central policy concern in many countries, including China (3). Healthcare resource misallocation is not unique to China. International evidence shows that financing, governance and payment incentives strongly shape how resources are distributed, with inefficient allocation of hospital capacity and specialist services worsening access and system performance in some high-income settings (4). Global reviews and case studies from low- and middle-income countries further stress that public financing and local governance critically determine whether reforms translate into more equitable and efficient resource allocation (5).

From the perspectives of classical sociology and social medicine, healthcare system reform is regarded as a critical pathway to address the misallocation of healthcare resources. The Social Determinants of Health (SDH) theory emphasizes that health is influenced not only by biological factors but also closely tied to social conditions (6). Reforming the healthcare system can enhance social conditions, particularly by improving healthcare resource allocation, thereby reducing health inequalities resulting from resource misallocation. Resource Dependence Theory posits that healthcare providers are constrained by the external resources they can access (7). Policy reforms can change resource flows by adjusting insurance payment rules and by strengthening support for grassroots providers, which in turn alters inter-hospital dependencies and reduces misallocation. Institutional Theory emphasizes that redesigning rules and governance arrangements can shift incentives and organizational behavior, mitigating excessive concentration of services (8). Path Dependence Theory highlights that historical institutional patterns shape current allocation and that deliberate institutional change is often required to break entrenched allocation dynamics (9). Thus, healthcare system reform, by altering institutional frameworks, optimizing resource allocation, and enhancing grassroots service capabilities, provides a theoretical foundation for addressing healthcare resource misallocation.

Existing research has predominantly concentrated on the impact of healthcare reform on the efficiency of resource allocation, yet it largely falls short of probing the causal nexus between reform and resource misallocation. For instance, comprehensive healthcare reforms have significantly improved resource allocation efficiency across Chinese provinces, particularly in eastern and central regions, but these studies have not delved into the specific mechanisms of healthcare resource misallocation. Similarly, while healthcare resource misallocation is more severe in underdeveloped regions of China, the impact of reforms on this phenomenon has not been thoroughly analyzed. In the United States, although certain reforms have improved emergency department resource misallocation, issues of resource shortages and demand misalignment persist, highlighting the uncertainty of reform outcomes. Additionally, while Indonesia has made progress in medical infrastructure development, the actual benefits of resource allocation have not been fully realized and are influenced by local political factors. Furthermore, while some scholars have emphasized the relationship between healthcare resource misallocation and regional disparities, their studies similarly lack causal identification and mechanism analysis. These findings indicate that while there is a substantial body of empirical research on the impact of healthcare reforms on resource allocation, there is a notable deficiency in exploring the specific mechanisms and causal effects of reforms on healthcare resource misallocation.

A substantial body of recent empirical research has evaluated the effects of healthcare reforms on resource allocation and related outcomes. For example, some studies document improvements in allocation efficiency following comprehensive reforms in several Chinese provinces (10, 11), while others report changes in emergency-department resource use in the United States (12); additional work highlights how political and implementation constraints can shape reform outcomes in settings such as Indonesia (13). However, relatively few studies directly treat city level resource misallocation as the primary outcome. In this paper city level resource misallocation is operationalized as a city s deviation from a national benchmark for per capita medical resources. Although prior work has made causal identification attempts and provided important evidence on efficiency, access and system performance (14–18), much of the literature focuses on allocation efficiency or service and output measures rather than on city level misallocation per se. Moreover, existing mechanism analyzes often rely on macro regressions or micro household and patient survey evidence rather than subjecting candidate mediators such as government health expenditure or insurance enrollee scale to separate synthetic control analyzes within the same quasi experimental framework that identifies the overall policy effect.

Motivated by these gaps, this paper examines city-level resource misallocation using Sanming’s 2012 healthcare reform as a quasi-natural experiment. We apply the synthetic control method to construct a long-run counterfactual for Sanming, subject key mediators to separate SCM analyzes, and perform multiple robustness checks to provide more direct and rigorous evidence on whether and how the reform reduced resource misallocation. Identifying the causal effects of healthcare reforms presents a significant challenge, particularly in the absence of randomized controlled trials. Nevertheless, the profound healthcare reform initiated in Sanming City, China, in 2012 offers an ideal quasi-natural experimental setting. As a pivotal case of healthcare reform in China, the reforms in Sanming City have been gradually rolled out nationwide, offering a valuable external policy shock for identifying the effects of healthcare reforms. This study will leverage the Sanming experiment, employing the cutting-edge synthetic control method (SCM) to discern the causal impact of healthcare reform on resource misallocation and to elucidate its latent mechanisms. Through this analysis, this study seeks to provide policymakers with empirical evidence regarding the specific impact of healthcare reforms on healthcare resource allocation and their mechanisms, thereby offering theoretical support and practical guidance for healthcare reforms in China and other countries, ultimately promoting more efficient and equitable healthcare resource allocation and enhancing the fairness and efficiency of global health systems.

Compared to the traditional difference-in-differences (DID) method, the SCM approach effectively overcomes the constraints imposed by the parallel trends assumption, rendering it particularly suitable for evaluating policy interventions in isolated regions. SCM delivers more precise counterfactual estimates while mitigating issues of spatial heterogeneity. Consequently, it is better suited than DID for identifying the causal effects examined in this study. Selecting China as the research context is justified firstly by the extensive and profound nature of its healthcare system reforms—especially given that local reforms produce significant externalities that offer an ideal policy experimental setting. Secondly, China faces acute challenges in the misallocation of medical resources, with marked regional disparities that make its experience both representative and widely instructive.

The remainder of this paper is structured as follows: Section Two provides an overview of the relevant policy background of the Sanming healthcare reforms; Section Three conducts theoretical analysis and proposes two hypotheses to be tested; Section Four outlines the research design; Section Five presents the empirical analysis results in detail; and Section Six engages in an extensive discussion with existing literature.

## 2 Policy background

Sanming is located in central Fujian Province, China and is an emerging industrial city whose economy has long been supported by sectors such as steel cement and heavy machinery. The city has long faced notable regional and urban rural disparities in healthcare allocation especially at the primary care level where shortages and limited service capacity persist. In response, Sanming initiated a profound healthcare reform in 2012—hereinafter referred to as “Sanming Medical Reform”—which has become one of the foremost exemplar cases of medical reform in China, offering invaluable lessons for nationwide application. The reform aimed to rebalance resource distribution strengthen primary care reduce pressure on large hospitals and establish a tiered diagnosis and treatment system. Sanming promoted referral to primary care strengthened grassroots infrastructure and introduced family doctor contract services to improve prevention reduce redundant treatments and support service capacity. The reform also restructured public hospital management including a slimming plan to rebalance beds and staffing and measures to improve efficiency and reduce patient costs. Insurance payment reforms expanded reimbursement and encouraged payment innovations to support universal coverage. Challenges remain because building primary care capacity takes time and investment; early on many patients still preferred large hospitals and tertiary institutions continue to face pressure, highlighting the difficulty of balancing distribution while maintaining quality.

Sanming’s reform has not only provided a viable model for other regions in China but has also offered valuable insights to the international community. By optimizing the distribution of healthcare resources, the reform has alleviated overcrowding in major hospitals and enhanced the accessibility and quality of primary care services. The practical experience of Sanming illustrates that tiered healthcare systems and resource optimization can effectively address the issue of excessive concentration, thereby improving overall service efficiency. Nevertheless, the implementation of such reforms must be attuned to the distinct economic and social contexts of each locality, necessitating bespoke adaptations for broader application. In summary Sanming’s reform addressed resource wastage and inequitable access and offers practical guidance for reforms elsewhere while underscoring the ongoing challenge of balancing equity and efficiency in health system design.

## 3 Mechanism hypothesis

### 3.1 Increase government healthcare expenditure

Public finance theory posits that governmental spending can remedy market failures and enhance resource allocation efficiency. In the healthcare market, information asymmetry and supply-side imbalances often result in an overconcentration of resources in large hospitals, while primary care facilities, hampered by limited funding and outdated infrastructure, remain underutilized. Augmenting government expenditure not only bolsters the infrastructure and service capacity of grassroots institutions—thereby enhancing their appeal to patients—but also improves the remuneration of primary care professionals, ultimately rectifying the supply–demand mismatch and curtailing resource misallocation. Moreover, transaction cost theory suggests that misallocation arises not only from imbalances in supply and demand but also from the costs incurred by patients seeking care. More importantly, increased government healthcare expenditure directly alleviates resource misallocation by shifting both financial and human resources toward primary care institutions, thereby weakening the structural overconcentration in tertiary hospitals. Higher spending reduces the dependency of large hospitals on excessive patient inflows while expanding the service capacity of grassroots providers, which in turn channels patient demand in a more balanced manner across the system. Thus, the increase in healthcare expenditure is not only an input of financial resources but also a mechanism that systematically realigns the distribution of medical resources (19). In summary, the Sanming Medical Reform has achieved a rational allocation of healthcare resources by reinforcing primary care capacity on the supply side, refining the insurance payment system on the demand side, and lowering patients’ overall cost of care.

*Hypothesis 1:* The Sanming health care reform effectively reduces the mismatch of health care resources by increasing government healthcare expenditures.

### 3.2 Improved enrollment targeting and efficiency

Within the traditional health insurance framework, a subset of enrollees may redundantly subscribe or inefficiently exploit insurance benefits due to lax policy oversight, occasionally resulting in overmedicalization and the unwarranted consumption of insurance funds—thereby leaving genuinely needy patients inadequately protected. The Sanming Medical Reform addresses this inefficiency by eliminating duplicate enrollments and rigorously verifying eligibility, thus enhancing the efficiency of insurance fund utilization and ensuring that resources are accurately directed toward those in need. As a consequence, the reform inevitably reduced the number of recorded insured individuals. However, this reduction does not indicate a weakening of coverage; rather, it reflects the removal of invalid, overlapping, or fraudulent enrollments. By cleansing the insurance pool, the reform freed up funds that could be reallocated to patients with genuine medical needs. In this sense, the decrease in the number of insured is theoretically linked to the alleviation of resource misallocation, as insurance resources are no longer dissipated across false or redundant beneficiaries but instead concentrated on the population truly requiring protection (20). These measures reduced recorded enrollee counts primarily by removing duplicate and ineligible entries and by improving registry quality, and thereby they improved the efficiency of fund use and helped direct resources to patients who truly need them in a manner consistent with the universal coverage principle of China s medical insurance policy (21). Furthermore, by revising insurance payment methods and increasing reimbursement ratios for primary care institutions, the reform diminishes the overreliance of major hospitals on insurance funds, encourages appropriate patient triage based on illness severity, and alleviates resource overload in large hospitals, thereby boosting the utilization of grassroots services. This process aligns with the Demand–Supply Matching Theory, which contends that the equitable distribution of healthcare resources should reflect true medical needs rather than an overabundance of insurance funds or misguided incentives.

*Hypothesis 2:* The Sanming health care reform effectively reduces the misallocation of health care resources by improving enrollment targeting and efficiency.

## 4 Research design

### 4.1 Data sources and data processing

The raw dataset was constructed from the National Bureau of Statistics, municipal statistical bureaus, annual City Statistical Yearbooks, and local health commission websites. To ensure data integrity and comparability across cities, we adopted the following rules: (i) cities with missing dependent variable values or insufficient observation years were entirely excluded, yielding a balanced panel of 204 cities from 2007 to 2022; (ii) for the mechanism variable “number of health insurance participants,” which was only available from 2011 onwards, missing pre-2011 values were imputed using linear interpolation to maintain continuity and allow application of the synthetic control method. This treatment is consistent with established practices in panel data analysis.

### 4.2 Synthetic control method

The synthetic control method is a quantitative technique designed to evaluate the impact of policy interventions or events, particularly in contexts where randomized controlled trials are impractical. Its core premise involves constructing a weighted control group that mirrors the treated unit’s pre-intervention characteristics, thereby simulating its counterfactual trajectory absent the policy intervention. In this study, SCM is employed to assess the effect of Sanming’s healthcare reform on medical resource misallocation. The methodology involves: firstly, selecting 203 cities unaffected by the policy as the control group; secondly, using an optimization algorithm to assign weights to these cities so that their aggregate pre-intervention characteristics—across economic, social, and other relevant dimensions—closely match those of Sanming; thirdly, estimating the counterfactual trend of healthcare resource misallocation for Sanming in the absence of reform; and finally, quantifying the reform’s impact by comparing the actual post-reform outcomes with the synthetic control’s estimated trajectory. For detailed mathematical derivations, see Abadie et al. (22, 23).

Compared to traditional DID methods, SCM has significant advantages. While DID assumes that the treated and control groups have identical trends before the intervention, this assumption often fails in practice due to substantial differences in economic and social structures across cities. SCM circumvents this limitation by constructing a synthetic control group that closely matches the treated city in terms of its pre-intervention characteristics, providing a more precise counterfactual estimation. Additionally, SCM can address potential spatial heterogeneity issues, making it particularly suitable for evaluating localized policy interventions like the Sanming healthcare reform, which may involve significant geographic and economic disparities. Therefore, SCM offers a more accurate and stable tool for assessing policy effects, providing valuable support for evaluating the outcomes of reforms at the local government level.

### 4.3 Variable definitions

#### 4.3.1 Dependent variable

The misallocation of medical resources, serving as the dependent variable, is quantified by measuring the deviation from the national average (24). This approach was selected because it offers a parsimonious and interpretable measure that facilitates comparisons across regions and time. It also aligns with prior studies that have used national benchmarks as reference values in evaluating allocation disparities. This is achieved through the following steps: (1) normalizing and summing the city-level counts of hospitals and health centers, hospital beds, and physicians to obtain the total medical resources for each city; (2) calculating the annual national average of these resources; and (3) computing the misallocation value for each city using the formula (1):


(1) $${\text{Misallocation}}_{\text{city},\text{year}}=|\frac{\begin{array}{l} \text{medical}\_{\text{resources}}_{\text{city},\text{year}}\\ -\bar{\text{medical}\_{\text{resources}}_{\text{year}}} \end{array}}{\bar{\text{medical}\_{\text{resources}}_{\text{year}}}}|$$

**Figure 1:**
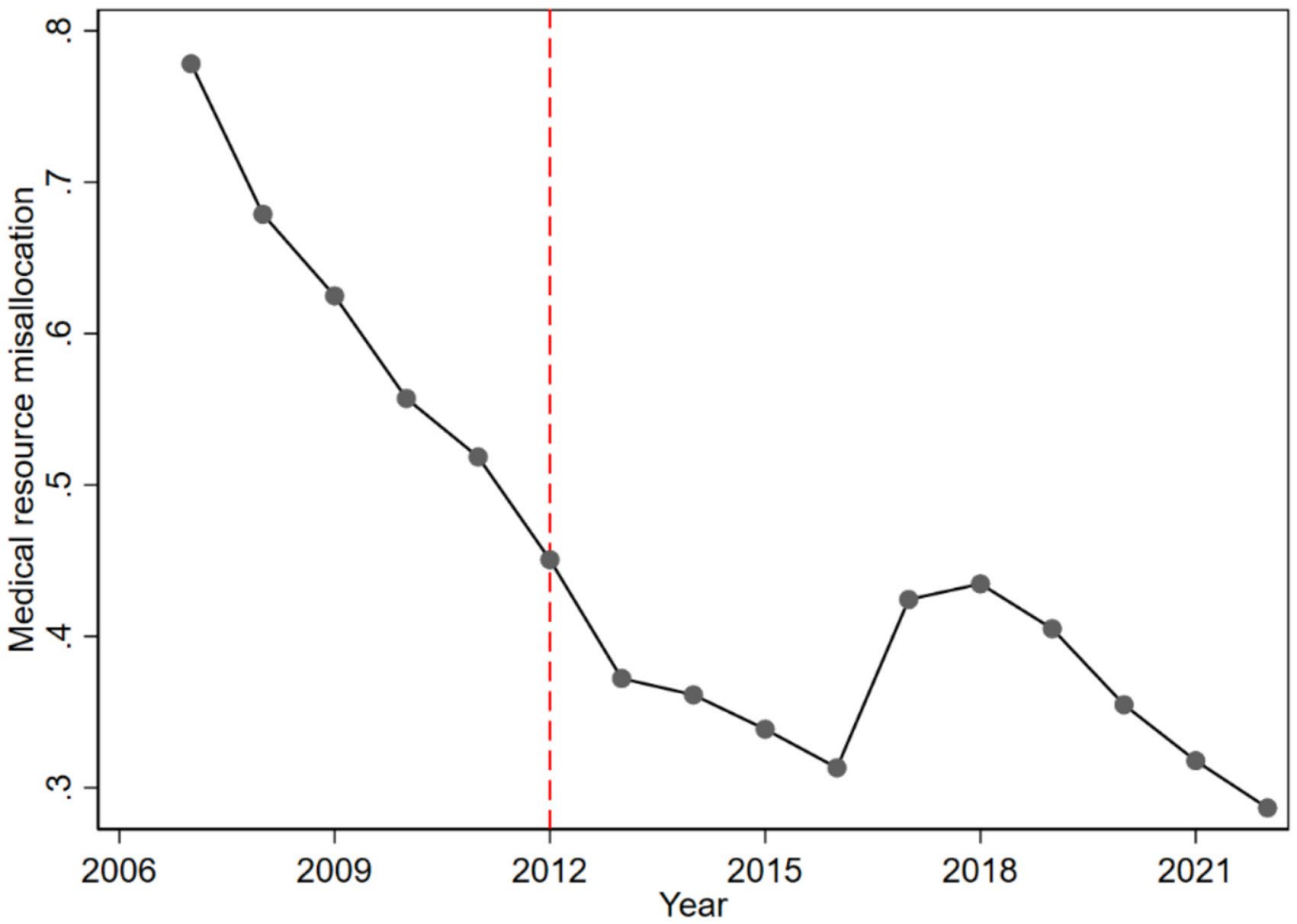
Time trend of misallocation of medical resources in Sanming City.

Where: $\text{Misallocatio}n_{\text{city},\text{year}}$ represents the degree of medical resource misallocation for a city in a given year; $\text{medical}\_\text{resource}s_{\text{city},\text{year}}$ represents the total medical resources for a city in a given year; $\bar{\text{medical}\_\text{resource}s_{\text{year}}}$ represents the national average of medical resources for that year. Clearly, a higher value of $\text{Misallocatio}n_{\text{city},\text{year}}$ indicates a greater degree of medical resource misallocation. Figure 1 depicts the temporal trend of medical resource misallocation in Sanming, revealing an overall decline with a minor uptick during 2017–2018.

#### 4.3.2 Covariates

In SCM, covariates are instrumental in determining the optimal weighted combination of control cities to accurately reflect the pre-intervention characteristics of the treated unit. The covariates selected in this study include: the proportion of secondary industry value-added in GDP, reflecting the regional industrial structure; the proportion of tertiary industry value-added in GDP, reflecting the regional industrial structure; regional GDP (in ten thousand yuan), indicating the economic scale of the region; regional GDP growth rate (%), indicating the economic development trend of the region; registered population (in ten thousand), indicating the total human resources in the region. Additionally, to account for potential temporal dependencies or lag effects in healthcare resource misallocation, the average misallocation values from 2007, 2008, 2009, and 2010 are incorporated. This approach not only better captures dynamic effects and minimizes omitted variable bias but also enhances the fit between the treated unit and its synthetic control, thereby improving the accuracy of the counterfactual estimates. Table 1 presents the balance test results for these covariates, demonstrating minimal bias post-weighting and affirming a robust parallel trend prior to the policy intervention.

**Table 1:** Covariate balance between Sanming City and the synthetic control in the pretreatment period.

Covariate	V. weight	Treated	Synthetic control	Bias(%)
Percentage of secondary industry value-added	0.0005	47.288	46.9304	−0.76%
Percentage of tertiary industry value-added	0.0003	33.87	33.5704	−0.88%
GDP	0.0004	8.40E+06	8.14E+06	−3.15%
Household population	0.001	271.368	265.7329	−2.08%
GDP growth rate	0	14.04	13.9476	−0.66%
Misallocation (2010)	0.3756	0.5572	0.5479	−1.67%
Misallocation (2009)	0.2453	0.6249	0.6167	−1.32%
Misallocation (2008)	0.1921	0.6789	0.6724	−0.95%
Misallocation (2007)	0.1847	0.7782	0.7723	−0.76%

V. weight refers to the optimal diagonal weight of each covariate in the synthetic control model. The synthetic control is the weighted average of donor cities chosen to best replicate Sanming before the 2012 reform. Bias (%) measures the relative difference between Sanming and its synthetic control for each covariate, showing that pre-treatment characteristics are closely matched.

## 5 Results

### 5.1 Basic results

**Figure 2:**
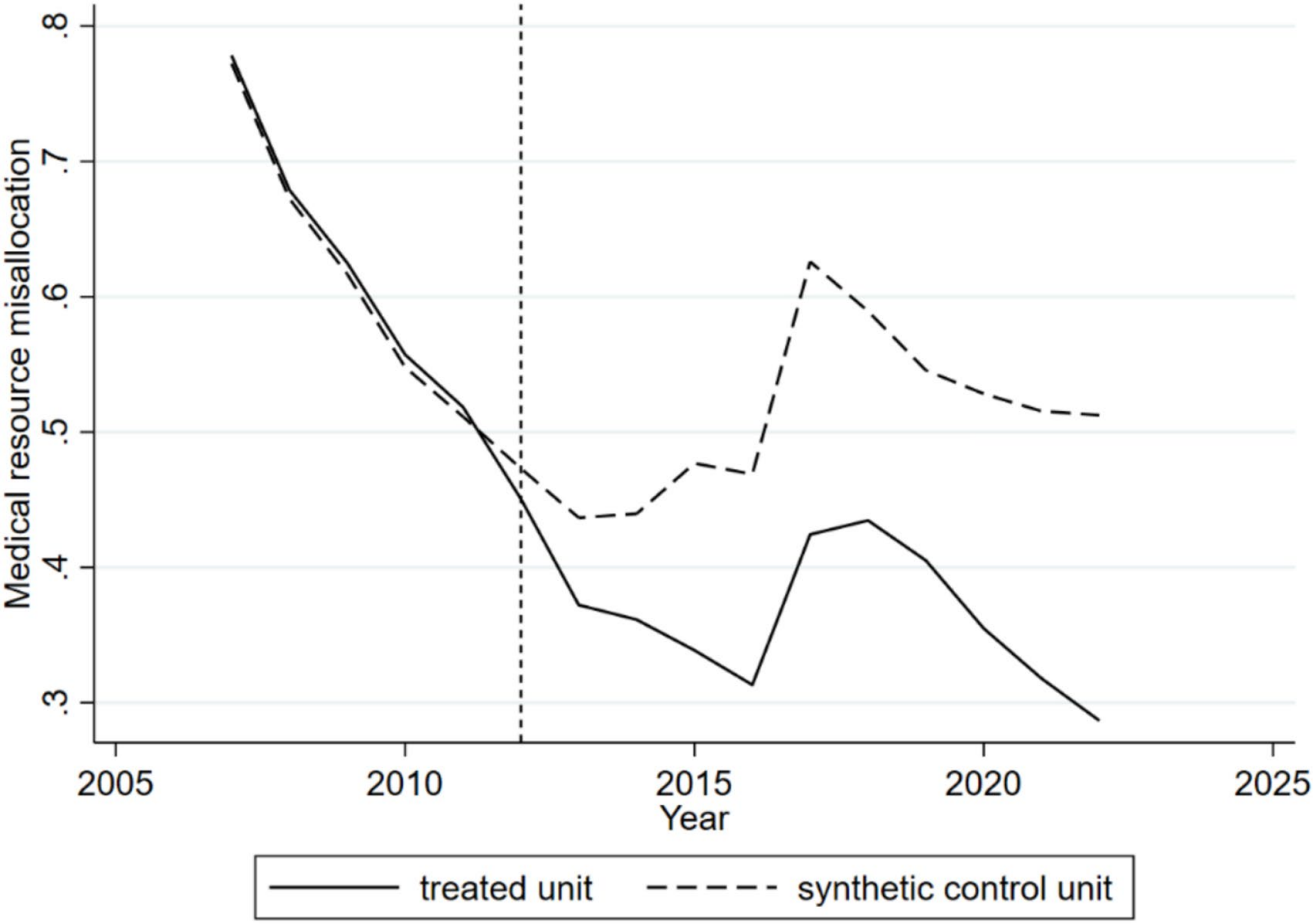
Dynamic effects of the Sanming Medical Reform on medical resource misallocation (synthetic control method, 2006–2019). The solid line shows Sanming’s actual trajectory, and the dashed line shows the synthetic control. The close overlap before 2012 confirms a valid counterfactual. After the reform, Sanming’s misallocation index remains consistently lower, with the widening gap indicating the reform’s mitigating effect.

Figure 2 illustrates the SCM outcomes. Prior to the 2012 shock of the Sanming Medical Reform, the dynamic trajectories of medical resource misallocation for Sanming (the treated unit) and the weighted aggregate of other cities (the synthetic control) were virtually indistinguishable, demonstrating a robust pre-intervention parallel trend. This affirms that the synthetic control aptly represents Sanming’s counterfactual scenario. Post-2012, a pronounced divergence emerges: while the synthetic control exhibits a transient rise followed by a decline in misallocation, Sanming’s curve, although displaying a similar pattern, consistently remains lower, with the gap between the two gradually widening. This divergence vividly underscores the significant mitigating effect of the reform on medical resource misallocation.

Table 2 presents the medical resource misallocation values (actual outcome) for Sanming City, the synthetic outcome for other weighted cities (synthetic outcome), and the difference between the two (treatment effect) after the policy shock. The treatment effect, which directly reflects the causal impact of the reform, shows that Sanming City’s misallocation value decreased steadily from −0.0225 in 2012 to −0.2016 by 2017. In 2018 and 2019, the policy effect weakened slightly, but it strengthened again from 2020 onward. On average, the Sanming healthcare reform reduced the city’s medical resource misallocation by 0.1412, highlighting its long-term effectiveness.

**Table 2:** Estimated treatment effects of the Sanming Medical Reform in the posttreatment period.

Year	Actual outcome	Synthetic outcome	Treatment effect
2012	0.4506	0.4731	−0.0225
2013	0.3722	0.4367	−0.0645
2014	0.3614	0.4396	−0.0783
2015	0.3387	0.4769	−0.1382
2016	0.3132	0.4688	−0.1556
2017	0.4243	0.6259	−0.2016
2018	0.4347	0.5893	−0.1546
2019	0.4051	0.5456	−0.1406
2020	0.3549	0.5282	−0.1734
2021	0.3179	0.5154	−0.1976
2022	0.2867	0.5125	−0.2258
Mean	0.369	0.5102	−0.1412

“Actual outcome” denotes the observed index of medical resource misallocation in Sanming City, while “Synthetic outcome” is the counterfactual trajectory estimated by the synthetic control. The “Treatment effect” is defined as the difference between the two. The average effect across 2012–2022 is −0.1412, indicating a sustained reduction in misallocation following the reform.

Table 3 reports the donor city weight distribution used to construct the synthetic Sanming, showing each control city’s contribution to the synthetic counterfactual.

**Table 3:** Donor city weight distribution for constructing the synthetic Sanming.

No.	City	Effect	No.	City	Effect	No.	City	Effect	No.	City	Effect
1	Shijiazhuang	0.000	52	Yangzhou	0.001	103	Weifang	0.000	154	Heyuan	0.001
2	Tangshan	0.000	53	Zhenjiang	0.001	104	Jining	0.000	155	Qingyuan	0.001
3	Hohhot	0.002	54	Taizhou	0.001	105	Tai’an	0.002	156	Zhongshan	0.005
4	Baotou	0.002	55	Suqian	0.001	106	Weihai	0.034	157	Yunfu	0.001
5	Wuhai	0.180	56	Hangzhou	0.039	107	Rizhao	0.002	158	Nanning	0.000
6	Chifeng	0.001	57	Ningbo	0.001	108	Linyi	0.000	159	Liuzhou	0.003
7	Tongliao	0.002	58	Wenzhou	0.000	109	Dezhou	0.001	160	Guilin	0.002
8	Ordos	0.008	59	Jiaxing	0.002	110	Liaocheng	0.002	161	Wuzhou	0.001
9	Hulunbuir	0.003	60	Huzhou	0.002	111	Binzhou	0.002	162	Beihai	0.001
10	Bayannur	0.003	61	Shaoxing	0.001	112	Heze	0.000	163	Fangchenggang	0.000
11	Ulanqab	0.003	62	Jinhua	0.001	113	Kaifeng	0.001	164	Qinzhou	0.000
12	Shenyang	0.000	63	Quzhou	0.002	114	Luoyang	0.001	165	Guigang	0.001
13	Dalian	0.000	64	Zhoushan	0.001	115	Pingdingshan	0.002	166	Yulin	0.001
14	Anshan	0.001	65	Taizhou	0.001	116	Anyang	0.002	167	Hezhou	0.002
15	Fushun	0.001	66	Lishui	0.002	117	Hebi	0.003	168	Hechi	0.005
16	Dandong	0.002	67	Wuhu	0.001	118	Xinxiang	0.001	169	Laibin	0.003
17	Jinzhou	0.001	68	Bengbu	0.002	119	Jiaozuo	0.001	170	Haikou	0.004
18	Yingkou	0.002	69	Huainan	0.003	120	Puyang	0.002	171	Chengdu	0.000
19	Liaoyang	0.003	70	Ma’anshan	0.001	121	Xuchang	0.002	172	Zigong	0.002
20	Panjin	0.036	71	Huaibei	0.003	122	Luohe	0.001	173	Panzhihua	0.002
21	Tieling	0.003	72	Tongling	0.001	123	Sanmenxia	0.001	174	Luzhou	0.001
22	Chaoyang	0.002	73	Anqing	0.001	124	Nanyang	0.000	175	Deyang	0.003
23	Huludao	0.002	74	Huangshan	0.002	125	Shangqiu	0.022	176	Mianyang	0.001
24	Changchun	0.000	75	Chuzhou	0.001	126	Xinyang	0.001	177	Guangyuan	0.005
25	Jilin	0.001	76	Fuyang	0.001	127	Zhoukou	0.000	178	Suining	0.003
26	Siping	0.002	77	Suzhou	0.001	128	Zhumadian	0.001	179	Neijiang	0.001
27	Liaoyuan	0.001	78	Lu′an	0.001	129	Huanggang	0.001	180	Leshan	0.002
28	Tonghua	0.002	79	Bozhou	0.001	130	Changsha	0.000	181	Nanchong	0.001
29	Baishan	0.001	80	Chizhou	0.001	131	Zhuzhou	0.003	182	Meishan	0.001
30	Songyuan	0.001	81	Xuancheng	0.001	132	Xiangtan	0.003	183	Yibin	0.002
31	Harbin	0.000	82	Fuzhou	0.001	133	Hengyang	0.000	184	Guang’an	0.002
32	Qiqihar	0.004	83	Xiamen	0.004	134	Shaoyang	0.000	185	Dazhou	0.001
33	Jixi	0.002	84	Sanming		135	Yueyang	0.001	186	Ya’an	0.009
34	Hegang	0.005	85	Quanzhou	0.001	136	Changde	0.001	187	Bazhong	0.130
35	Shuangyashan	0.001	86	Nanchang	0.002	137	Zhangjiajie	0.001	188	Ziyang	0.002
36	Daqing	0.026	87	Jingdezhen	0.001	138	Yiyang	0.001	189	Guiyang	0.002
37	Yichun	0.002	88	Pingxiang	0.001	139	Chenzhou	0.002	190	Liupanshui	0.001
38	Jiamusi	0.002	89	Jiujiang	0.002	140	Yongzhou	0.001	191	Anshun	0.001
39	Qitaihe	0.000	90	Xinyu	0.001	141	Huaihua	0.001	192	Xi’an	0.000
40	Mudanjiang	0.003	91	Yingtan	0.001	142	Loudi	0.001	193	Lanzhou	0.002
41	Heihe	0.125	92	Ganzhou	0.001	143	Shaoguan	0.000	194	Jiayuguan	0.000
42	Shanghai	0.000	93	Ji’an	0.002	144	Shenzhen	0.000	195	Jinchang	0.000
43	Nanjing	0.000	94	Yichun	0.002	145	Zhuhai	0.004	196	Baiyin	0.002
44	Wuxi	0.001	95	Fuzhou	0.003	146	Shantou	0.001	197	Tianshui	0.004
45	Xuzhou	0.000	96	Shangrao	0.001	147	Foshan	0.001	198	Wuwei	0.011
46	Changzhou	0.001	97	Jinan	0.000	148	Jiangmen	0.001	199	Zhangye	0.088
47	Suzhou	0.000	98	Qingdao	0.000	149	Zhanjiang	0.001	200	Pingliang	0.002
48	Nantong	0.000	99	Zibo	0.003	150	Maoming	0.001	201	Jiuquan	0.003
49	Lianyungang	0.001	100	Zaozhuang	0.002	151	Zhaoqing	0.001	202	Qingyang	0.001
50	Huaian	0.001	101	Dongying	0.002	152	Huizhou	0.013	203	Dingxi	0.001
51	Yancheng	0.001	102	Yantai	0.000	153	Meizhou	0.005	204	Longnan	0.001

Sanming City (No. 84) is the treated unit and excluded from the donor pool. The synthetic control is a weighted average of 203 potential donor cities, with most cities assigned negligible weights. A small number of cities (e.g., Wuhai, Heihe, Bazhong, Zhangye) received relatively higher weights, indicating they contribute most to constructing the synthetic counterfactual.

### 5.2 City placebo test

**Figure 3:**
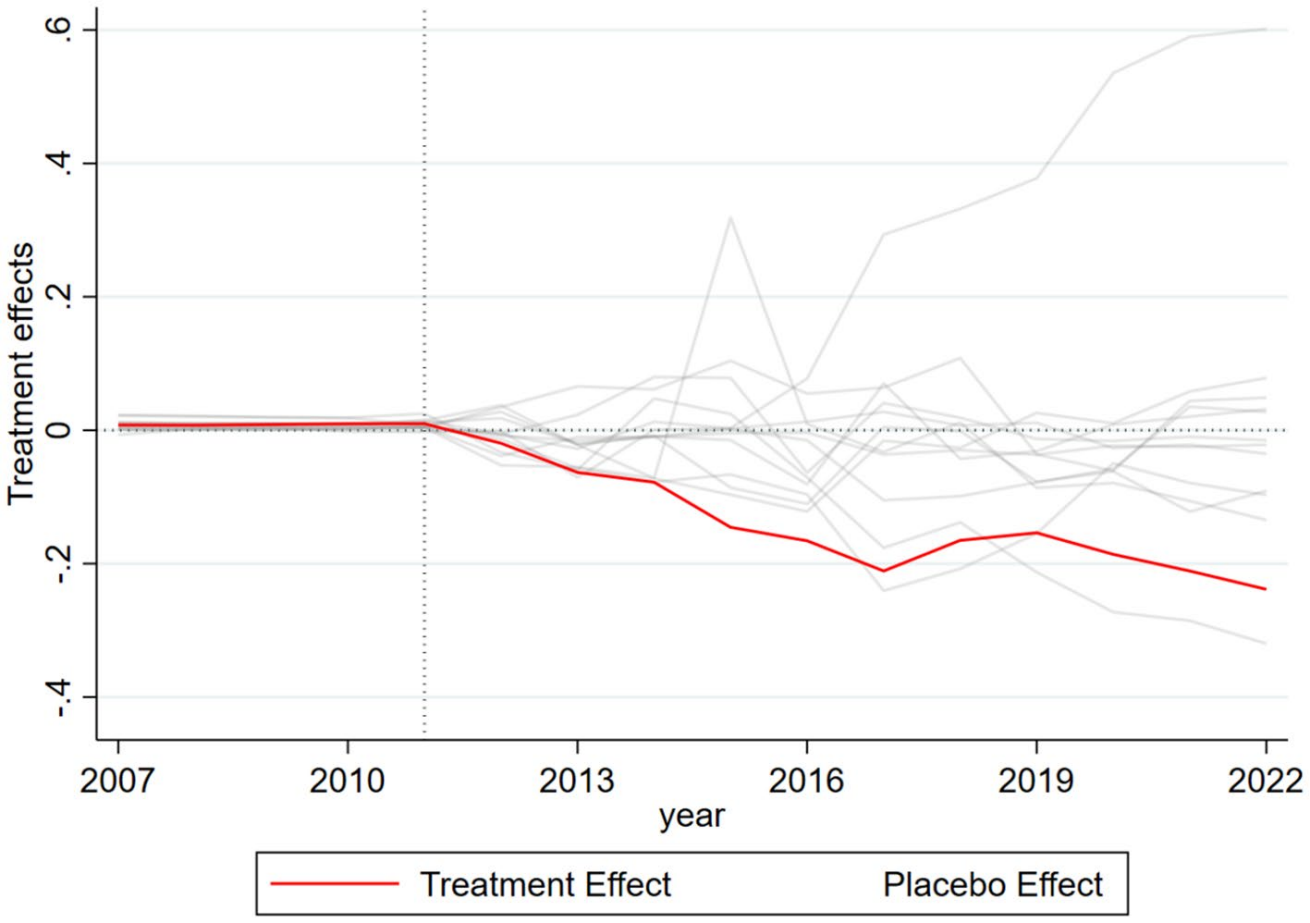
City-level placebo test results for the Sanming Medical Reform (synthetic control method). The red line depicts Sanming’s estimated treatment effect, while the gray lines represent 203 placebo tests assigning the reform to other cities. Most placebo curves lie above the Sanming curve, confirming that the observed effect is attributable to the reform rather than random assignment.

One concern with the SCM approach is whether the observed effect might stem from arbitrary group assignment rather than the true impact of the Sanming reform. To address this, we randomly designated alternative cities as placebo treatment units, generating 203 placebo effect curves. After excluding curves with an RMSE exceeding 700 times that of the actual curve (a less stringent threshold than the conventional fivefold, adopted here to retain a sufficient number of placebo curves), we obtained the following city placebo test results (Figure 3). The red curve represents the real policy effect (i.e., the treatment effect shown in Table 2), which is clearly distinct from the other placebo curves. The majority of the placebo curves remain above the real policy effect curve, statistically confirming that the observed results are indeed driven by the Sanming healthcare reform rather than random assignment or other confounding factors.

### 5.3 Time placebo test

**Figure 4:**
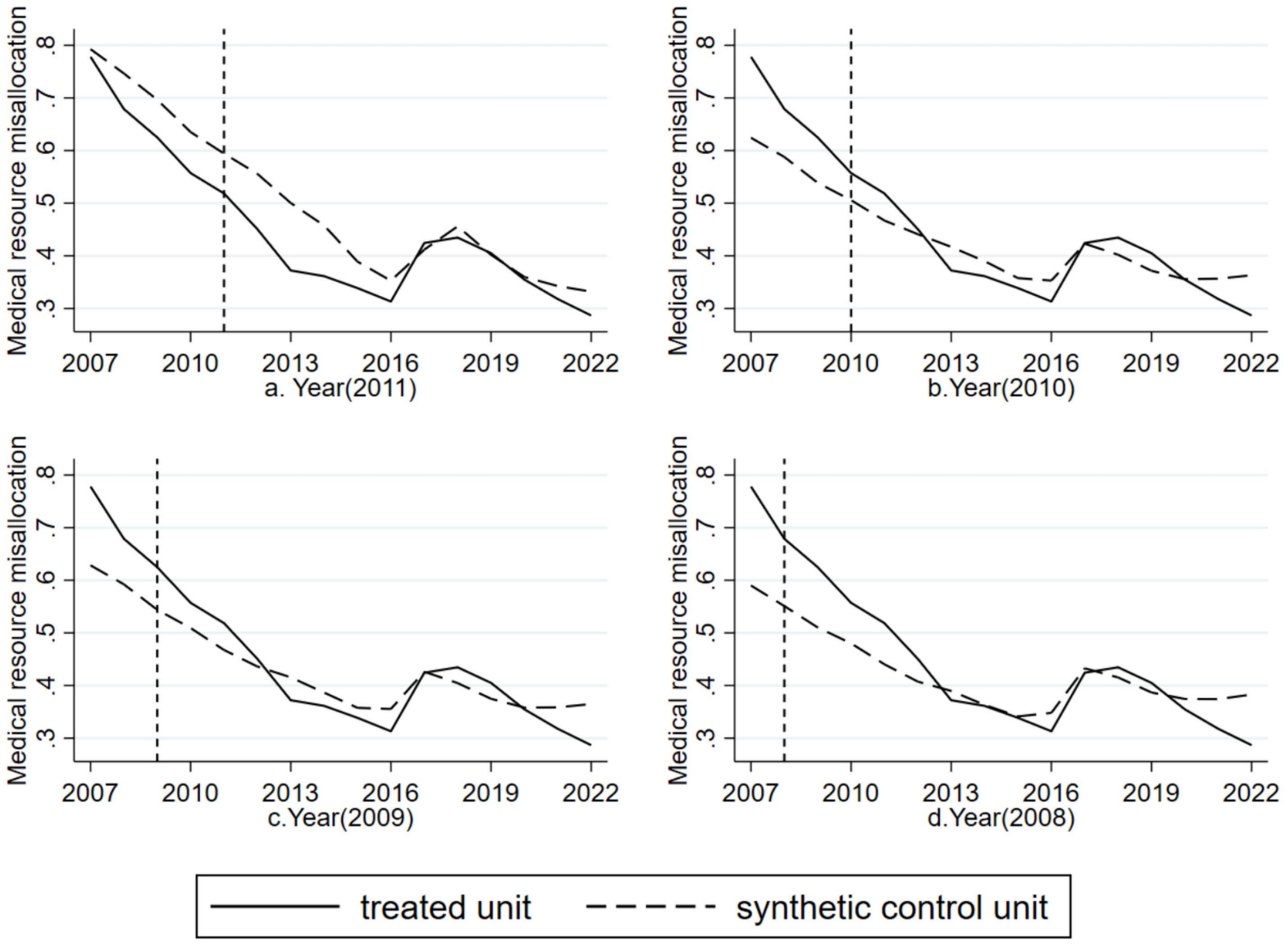
Time-placebo test results for the Sanming Medical Reform with artificially reassigned reform years. **(a)** Reform year reassigned to 2011; **(b)** reform year reassigned to 2010; **(c)** reform year reassigned to 2009; **(d)** reform year reassigned to 2008. In all cases, Sanming (treated unit) and its synthetic control unit showed no significant divergence, confirming that the observed effects in the main analysis are not driven by arbitrary policy timing.

Another concern is whether the observed policy effect might be due to the arbitrary timing of the policy implementation rather than its true impact. To address this, we randomly assigned policy implementation years to Sanming City before 2012 (specifically, 2011, 2010, 2009, and 2008) and generated four time placebo curves (Figure 4). Figure 4a shows the dynamics of medical resource misallocation if the reform had been implemented in 2011. The results demonstrate poor fit between Sanming City and the synthetic control unit before and after the policy shock, with no significant difference in trends. Figures 4b–d reflect similar analyzes for 2010, 2009, and 2008, respectively, with analogous results. Overall, these tests confirm that the policy effects observed in the original analysis are not due to the timing of the reform but rather its actual impact.

### 5.4 Robustness test

**Figure 5:**
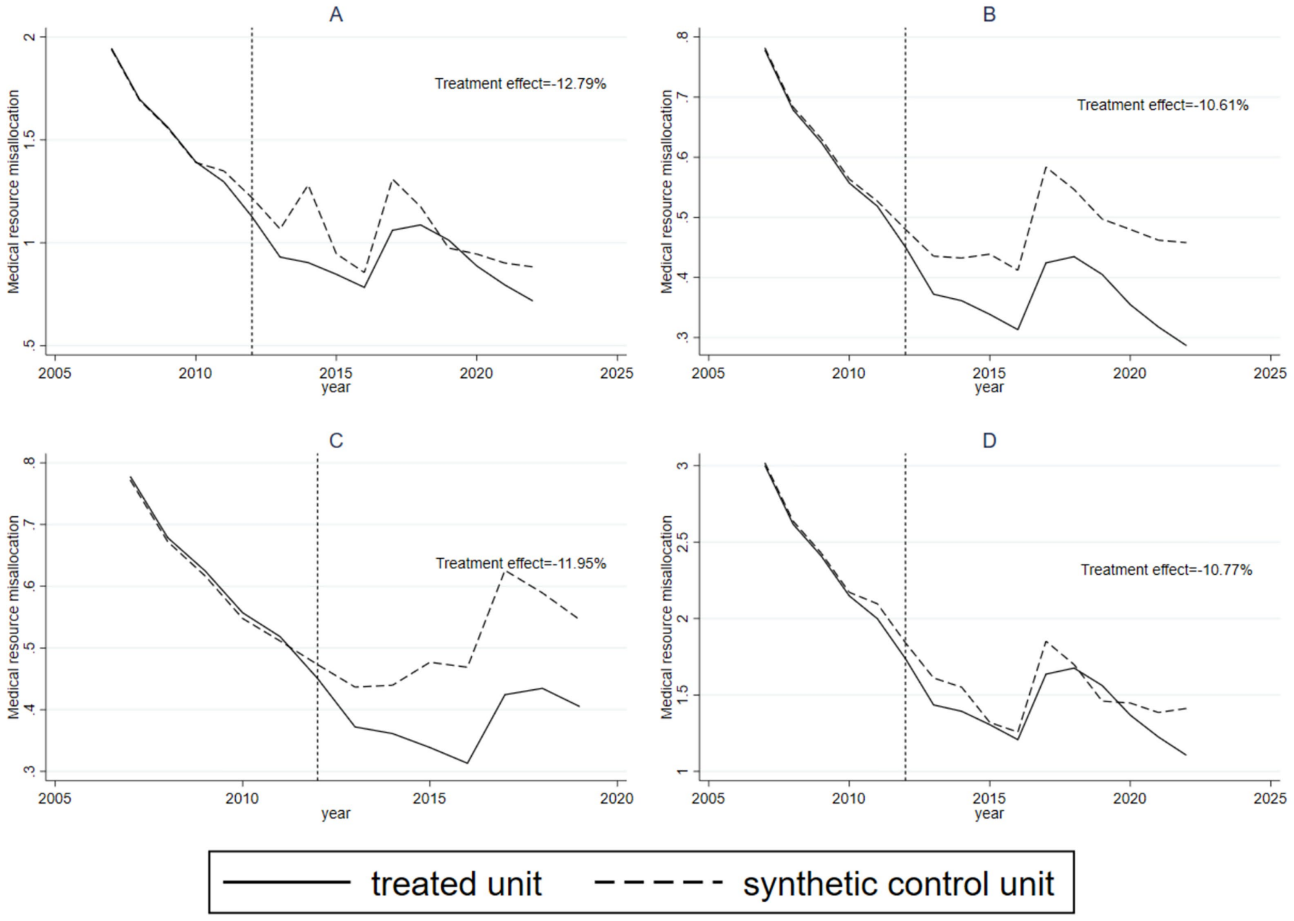
Robustness test results for the Sanming Medical Reform. **(A–D)** Report estimates after sequentially excluding **(A)** 2015–2016 national reform pilot provinces, **(B)** 2019 centralized drug procurement pilot cities, **(C)** post-2019 observations following nationwide promotion of the Sanming model, and **(D)** Fujian and neighboring provinces to address spatial spillovers. Across all scenarios, estimated effects remain close to the baseline, confirming the robustness of the findings.

In this section we undertake a series of robustness checks to affirm the stability of our principal findings. First, we exclude the eleven provinces and municipalities designated as comprehensive reform pilots by the State Council’s Medical Reform Leading Group in 2015–2016 (Shanghai, Zhejiang, Jiangsu, Anhui, Fujian, Hunan, Chongqing, Sichuan, Shaanxi, Qinghai, and Ningxia). These jurisdictions spearheaded reforms in insurance payment mechanisms, public hospital management innovation, and tiered care, and were later prioritized in 2021 as focal regions for advancing high-quality development of public hospitals; thus they may have systematically reshaped the supply and spatial distribution of medical resources and confounded identification of the Sanming effect. Re-estimating the synthetic control after excluding these regions (see Figure 5A) yields a policy effect of −12.79%, which is very close to the baseline estimate of −14.12%, indicating that our conclusions remain robust and that the Sanming reform produced a significant independent effect at the national level.

We further excluded the eleven cities designated in 2019 under the State Council’s pilot program for centralized drug procurement (Beijing, Tianjin, Shanghai, Chongqing, Shenyang, Dalian, Xiamen, Guangzhou, Shenzhen, Chengdu, and Xi’an). This procurement initiative achieved substantial price reductions through pooled purchasing of bioequivalent generics, alleviated patients’ financial burdens, lowered firms’ transaction costs, and catalyzed public hospital reform and the standardization of clinical prescribing; such systemic shifts could plausibly alter the allocation and utilization of medical resources and thus confound identification of the Sanming effect. Re-estimating the synthetic control after excluding these pilot cities yields a treatment effect of −10.61% (Figure 5B), which accords closely with the baseline result and supports the robustness of our findings.

We also considered the 2019 notice on promoting the Fujian and Sanming reform experience, which required provinces to develop implementation plans by the end of 2019 and led to the establishment of pilot evaluation and adjustment mechanisms in 2020. Because these measures facilitated nationwide replication of the Sanming model and could diminish contrasts between treated and donor units, we re-estimated the analysis using only samples through 2019; the resulting effect is −11.95% (Figure 5C), again consistent with the baseline estimate and reinforcing robustness.

Finally, to address potential spatial spillovers from the Sanming experiment to other prefectures in Fujian and to neighboring provinces (Zhejiang, Jiangxi, Guangdong), we excluded these regions from the donor pool and re-ran the estimation. The estimated treatment effect under this restriction is −10.77% (Figure 5D), which remains highly consistent with the primary results and further corroborates the independent impact of the Sanming reform.

### 5.5 Mechanism test

**Figure 6:**
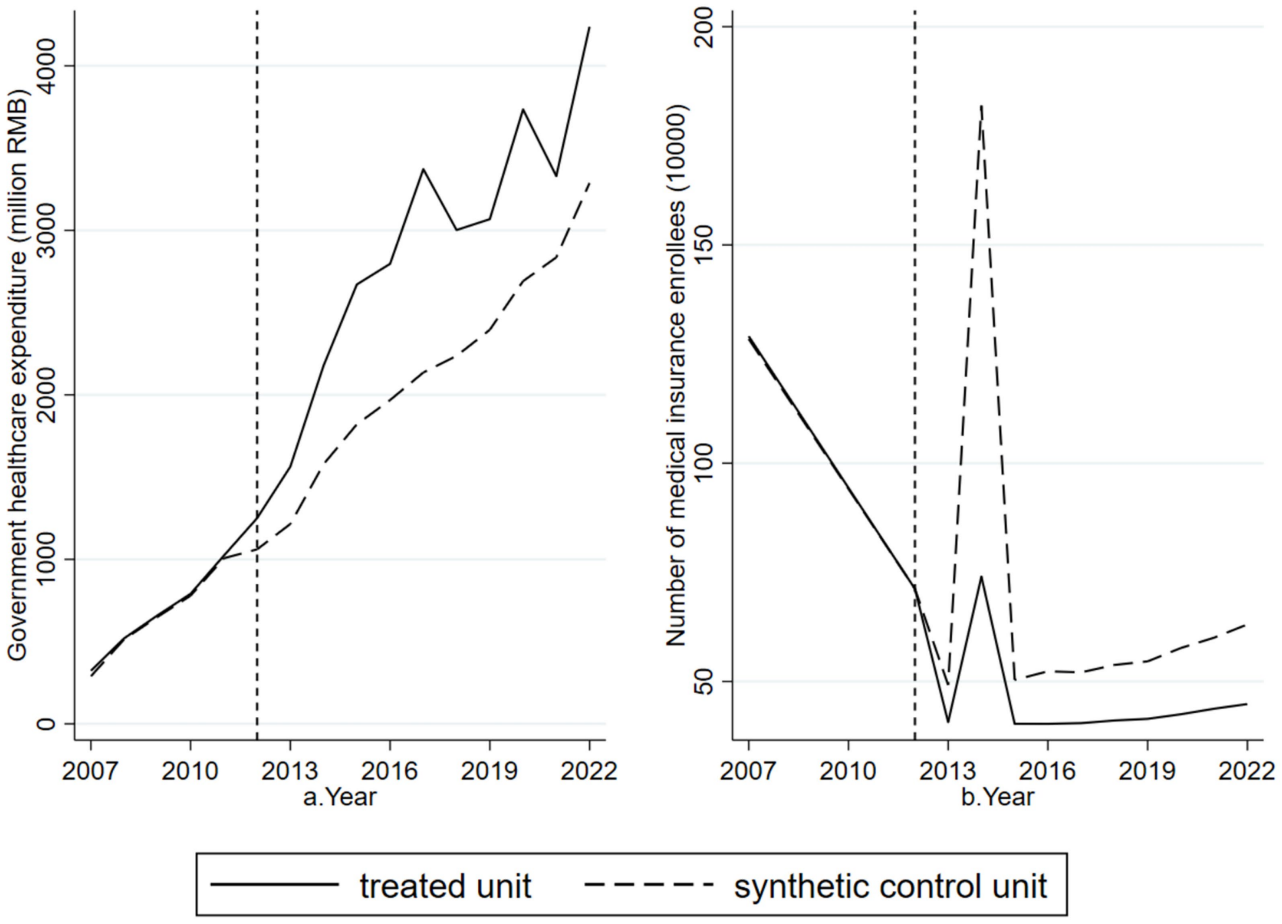
Mechanism test results for the Sanming Medical Reform. **(a)** Shows that government healthcare expenditure in Sanming rose markedly after 2012 compared with the synthetic control, supporting Mechanism Hypothesis 1. **(b)** Shows that the number of healthcare insurance participants declined relative to the control, supporting Mechanism Hypothesis 2. Together, these results illustrate the channels through which the reform reduced medical resource misallocation.

Figure 6a demonstrates that after the implementation of the Sanming healthcare reform in 2012, government healthcare expenditures in Sanming City increased significantly and remained higher than those in the synthetic control unit, supporting Mechanism Hypothesis 1. Figure 6b shows that after 2012, the number of healthcare insurance participants in Sanming City declined, significantly lower than in the synthetic control unit, supporting Mechanism Hypothesis 2. These results confirm the proposed mechanisms through which the reform influenced medical resource misallocation.

## 6 Discussion

The fundamental findings of this study indicate that the Sanming Medical Reform is associated with a substantial reduction in urban healthcare resource misallocation. Our analysis provides evidence that the reform in Sanming was followed by marked reductions in resource misallocation, a pattern that is consistent with existing literature. Sun et al. found that comprehensive healthcare reforms in China improved resource allocation efficiency, especially in eastern and central regions. This suggests that long-term improvements in distribution are attainable, which is consistent with the trajectory observed in Sanming (10). Deng et al. observed severe misallocation in many underdeveloped areas, while Sanming’s localized reform achieved effective reallocation and alleviated such imbalances (11). Wang and Jia reported a post-pandemic scarcity in primary care resources, whereas Sanming’s enhancement of grassroots capacity successfully mitigated misallocation (14). Dong and Wang discussed geographic disparities in healthcare resources, and Sanming’s strategy of resource decentralization contributed to a more equitable distribution across regions (15). Hsiao emphasized the pivotal role of local governments in resource distribution, and in Sanming, policy guidance effectively reduced imbalances (13). Chandra et al. highlighted how resource misallocation affects hospital productivity, and Sanming’s reform not only improved infrastructure but also enhanced service efficiency by reducing ineffective treatments (25). Collectively, these findings suggest that while the drivers of misallocation differ across contexts, the mechanisms of Sanming’s reform, including government stewardship, grassroots investment and payment reform, are aligned with international experiences. Global studies have highlighted targeted financing and governance as key levers for mitigating healthcare resource misallocation (5, 26). These empirical observations are consistent with the theoretical frameworks outlined in the Introduction. From the perspective of Social Determinants of Health (SDH), Sanming’s reforms improved equity by reallocating resources to grassroots institutions and vulnerable populations. In line with Institutional Theory, the reform reshaped governance and payment rules, reducing unnecessary concentration of services in tertiary hospitals. Consistent with Path Dependence Theory, the reform disrupted entrenched patterns of resource allocation, introducing institutional innovations that altered long-standing allocation dynamics. This suggests that Sanming’s progress provides policy lessons of wider relevance beyond the Chinese setting. At the same time, we recognize that the external validity of these findings should be interpreted with caution. Their applicability is likely to depend on a range of contextual conditions, including local institutional capacity and governance arrangements, the availability of fiscal space and the design of public financing and payment mechanisms, the baseline strength of primary care systems and the health workforce, and the political will to implement administrative measures such as enrollee verification and reimbursement reform. In contexts that differ substantially along these dimensions, the magnitude or even the direction of effects may differ.

The Sanming reform significantly reduced urban medical resource misallocation by increasing government healthcare expenditure, a finding consistent with existing research. Hosseini explored resource misallocation in emergency departments in the United States and noted that although reforms improved service delivery models, misallocation remained a systemic challenge (12). Sanming’s experience is consistent with this evidence by showing that increased government investment can mitigate inefficiencies, particularly when directed to primary care facilities. Hsiao studied misallocation in Indonesia’s healthcare infrastructure investment and found that local government bias in resource allocation exacerbated imbalances (13). In contrast, Sanming’s reform avoided such biases by directing resources to grassroots and underserved areas, thereby optimizing allocation. She et al. examined resource misallocation in the United States Medicare Advantage program and found that cross-subsidization worsened allocation inequities (27). Sanming’s reform, however, optimized expenditure allocation to ensure equitable distribution, particularly between grassroots hospitals and large hospitals. Amelung emphasized the importance of effective government expenditure allocation in maintaining healthcare supply in areas with limited human resources (28). Sanming’s case reflects this principle by demonstrating how targeted fiscal strategies improved allocation efficiency. Rosner discussed balancing resource allocation under constrained medical resources, and Sanming’s reform illustrated that strategic investment can improve both equity and efficiency (29). These cross-country comparisons reinforce the idea that although institutional designs differ, the challenges of resource misallocation are widely shared, and Sanming provides a concrete example of how targeted government action can alleviate them.

The Sanming reform was associated with a reduction in city-level medical resource misallocation, and our decomposed analyzes suggest this effect operated in substantial part through improvements in the targeting and efficiency of insurance enrollment and fund utilization. The slight weakening of the policy effect in 2018–2019 may reflect national replication of similar reforms, local economic fluctuations, and early COVID-19 preparedness. Although these minor fluctuations occurred, the overall downward trend and persistent improvements in fund targeting and primary care capacity indicate that the reform’s long-term effectiveness remains robust. Our research found that the Sanming medical reform led to a fluctuating decline in the number of people participating in medical insurance in Sanming City. Administrative measures such as the removal of duplicate or ineligible registrations and strengthened eligibility verification together with reimbursement adjustments that support primary care appear to have reduced recorded enrollee counts while improving access for eligible individuals and directing financial resources more toward those most in need. These changes may have increased the precision of fund allocation and helped shift utilization away from unnecessary tertiary care, thereby alleviating some resource concentration. This mechanism is consistent with previous studies that emphasize the role of governance and payment incentives in shaping resource distribution (25, 28–30). The way these measures were implemented also supports the universal coverage principle because they help ensure that insurance funds are used more effectively to protect genuinely needy populations. Previous studies have shown that tiered diagnosis and treatment reforms improved service accessibility but exacerbated inter-regional resource allocation imbalances (31). However, by refining enrollee numbers, Sanming has fostered the development of primary care, alleviating the strain on major hospitals and promoting a more balanced regional resource distribution.

Despite the compelling empirical evidence provided by the Sanming case, this study has some limitations. First, our primary misallocation measure is based on deviations from national averages in hospitals, beds and physicians. This parsimonious indicator supports comparability across cities and long run synthetic control analysis, but it does not capture other important dimensions such as service utilization, care quality and patient outcomes and may obscure within city heterogeneity. Second, although this study has explored the primary mechanisms underlying the reform, the specific causal pathways warrant further investigation, particularly with regard to differential impacts across regions and population segments. Third, we also acknowledge that misallocation is inherently multidimensional, and alternative approaches could provide richer insights. Due to data limitations, we adopted deviations from national averages as a parsimonious and comparable measure, but future studies may extend this work using more comprehensive approaches. Finally, while the Sanming experience offers useful lessons, its generalizability should be assessed in light of local institutional, fiscal and political contexts. The indicators employed in this study are indirect proxies and may not fully capture the causal mechanisms.

## Data Availability

The original contributions presented in the study are included in the article/supplementary material, further inquiries can be directed to the corresponding authors.
